# Bovine spongiform encephalopathy: the effect of oral exposure dose on attack rate and incubation period in cattle – an update

**DOI:** 10.1186/1756-0500-5-674

**Published:** 2012-12-05

**Authors:** Timm Konold, Mark E Arnold, Anthony R Austin, Saira Cawthraw, Steve AC Hawkins, Michael J Stack, Marion M Simmons, A Robin Sayers, Michael Dawson, John W Wilesmith, Gerald AH Wells

**Affiliations:** 1 Specialist Scientific Support Department, Animal Health and Veterinary Laboratories Agency Weybridge, New Haw, Addlestone, UK; 2 Oak Farm, Harpsden Bottom, Henley-on-Thames, UK; 3 TSE Department, Animal Health and Veterinary Laboratories Agency Weybridge, New Haw, Addlestone, UK; 4 Barton, 1 Woodham Road, Woking, UK; 5 Formerly - Consultant Veterinary Pathologist, Veterinary Laboratories Agency, New Haw, Addlestone, Surrey, KT15 3NB, UK

**Keywords:** Bovine spongiform encephalopathy, BSE, Cattle, Oral dose, Dose–response, Attack rate, Incubation period, Model, Risk of infection, Prion protein gene

## Abstract

**Background:**

To provide information on dose–response and aid in modelling the exposure dynamics of the BSE epidemic in the United Kingdom groups of cattle were exposed orally to a range of different doses of brainstem homogenate of known infectious titre from clinical cases of classical bovine spongiform encephalopathy (BSE). Interim data from this study was published in 2007. This communication documents additional BSE cases, which occurred subsequently, examines possible influence of the bovine prion protein gene on disease incidence and revises estimates of effective oral exposure.

**Findings:**

Following interim published results, two further cattle, one dosed with 100 mg and culled at 127 months post exposure and the other dosed with 10 mg and culled at 110 months post exposure, developed BSE. Both had a similar pathological phenotype to previous cases. Based on attack rate and incubation period distribution according to dose, the dose estimate at which 50% of confirmed cases would be clinically affected was revised to 0.15 g of the brain homogenate used in the experiment, with a 95% confidence interval of 0.03–0.79 g. Neither the full open reading frame nor the promoter region of the prion protein gene of dosed cattle appeared to influence susceptibility to BSE, but this may be due to the sample size.

**Conclusions:**

Oral exposure of cattle to a large range of doses of a BSE brainstem homogenate produced disease in all dose groups. The pathological presentation resembled natural disease. The attack rate and incubation period were dependent on the dose.

## Findings

### Background

To provide information on dose–response, minimum infective dose and aid in modelling the exposure dynamics of the BSE epidemic in the United Kingdom (UK) a study was conducted to determine the attack rate and dose response of cattle orally exposed to a range of different doses of classical bovine spongiform encephalopathy (BSE) brainstem homogenate of known infectious titre [1]. Although interim findings of this study have been published [1], the data presented for the second phase of the study were incomplete because of survival of some dosed cattle at the time (December 2006). The objective of this update is to present the completed data following termination of the second phase of the study, to document additional sequencing of the bovine prion protein gene (*PRNP*) of the studied cattle, with a possible influence on the incidence of disease, and to introduce a minor revision of the dose–response model given the occurrence of further BSE cases among recipients.

## Methods

All procedures involving cattle were approved under the UK Animal (Scientific Procedures) Act 1986 under licence from the Home Office of the UK government.

### Inoculum, animals and experimental design

The experimental design has been described in detail previously [1]. Briefly, groups of calves were dosed orally with a pool of BSE brainstem homogenate: 100 g on three consecutive days or single doses of 100 g, 10 g or 1 g (first phase) and 1 g, 100 mg, 10 mg, or 1 mg (second phase). The end point titre of the BSE brainstem homogenate, assayed in RIII mice, was 10^3.5^ mouse intracerebral/intraperitoneal (i.c./i.p.) ID_50_/g.

### Clinical monitoring

Clinical assessment methods, which varied between the study phases because of operational differences, have been described previously [1]. As previously the clinical status was categorised as possible, probable or definite signs of BSE [2] by the same assessor to allow comparability of study phases.

### Cull and postmortem examinations

Animals were culled after the display of definite signs of BSE, other untreatable conditions that required euthanasia or from 144 months post exposure (mpe), which was the study end-point. This end-point was estimated to encompass all incubation periods (IPs = time from exposure to display of definite signs of BSE or death whichever occurred earlier) with 95% probability based on interim data analysis of the IPs of all BSE cases in this study, which suggested that the IP would follow a log-normal distribution depending on dose (see below).

Postmortem examinations, including histopathology and immunohistochemical examination for the presence of disease-associated prion protein (PrP^d^), were as described previously for the second phase of the study [1].

Western immunoblotting (WB) for the proteinase-resistant form of the prion protein (PrP^res^) was previously carried out with the Prionics-Check Western technique (Prionics AG, Schlieren, Switzerland) [3], but was replaced for the present study with the BioRad TeSeE Western blot test (BioRad Laboratories, Marnes-La-Coquette, France) using the mAb Sha31 (BioRad Laboratories) [4] instead of mAb 6H4 (Prionics AG).

### Genotyping

Analysis of the bovine *PRNP* of the cattle was extended to include the promoter region and full open reading frame (ORF) from EDTA blood samples (live animal), semitendinosus muscle or brain (culled animal). DNA was extracted and amplified by polymerase chain reaction (PCR) as published previously [5] to determine bovine ORF polymorphisms and compare it to a wild-type PrP gene reference sequence from a Jersey cow, GenBank accession number AJ298878 [6].

To detect bovine PrP promoter 23 and 12 base pair (bp) insertions and deletions (indels), which have been investigated in association with BSE incidence [7], a TaqMan allelic discrimination assay was designed, optimised and performed, for each indel based on guidelines set out for designing TaqMan probes and primers (TaqMan Universal PCR Master Mix Protocol, Applied Biosystems, Foster City, USA). Forward and reverse PCR primer were 5'-CGGTTTTACCCTCCTGGTTAGG-3' and 5'-CCACCCACATACCTCGGG-3' for genotyping the 12-bp indel and 5'-GCCCAGGTGCCAGCC-3' and 5'-AAGAGTTGGACAGGCACAATGG-3' for the 23-bp indel. Fluorogenic probe (Applied Biosystems) sequences were 5’-CGGCCCCCGCCCACATTC-3’ and 5’-ACCAGCCAGCCCACATTCCGAGTAA-3’ for the 12-bp indel as well as 5’-AATCTCAGATGTCTTCCCAACAGCAGCC-3’ and 5’-CCTAGCTATCACGTCAAGCCTCAGACG-3’ for the 23-bp indel. The data were analysed by Sequence Detector Software (SDS) version 1.9.1 (Applied Biosystems).

The promoter and ORF regions of *PRNP* were assessed in 26 cattle from the first phase of the study for which tissue was available for testing [seven BSE-positive and three BSE-negative (at study termination at 100 mpe) cattle dosed with 1 g, five BSE-positive and two BSE-negative (at study termination at 100 mpe) cattle dosed with 10 g and nine BSE-positive cattle dosed with 100 g] and all 60 cattle from the second phase of the study.

### Effect of dose and genotype on probability of infection and incubation period

Data on the IP for each animal exposed to single doses and on the survival of the non-affected animals were used to estimate the dependence of both the IP distribution and attack rate on the dose, via maximum likelihood. Based on the assumption that the IP followed a log-normal distribution, with the mean depending linearly on the log dose, *d*, and that the attack rate followed a logistic regression curve, the maximum likelihood estimates of the parameters for the IP distribution and probability of infection according to dose (expressed in terms of mouse i.c./i.p. ID_50_) could be calculated using previously published formulae [1].

Proportional hazard (Cox) regression (Statistica version 10, StatSoft Inc., Tulsa, USA) was used to evaluate the effect of genotype and dose of inoculum on the survival of cattle inoculated with the BSE agent. The dependent variable was the time of cull/onset of definite signs, whereas independent variables were the dose (as log_10_), the octapeptide repeats (6:6 compared to others), the silent PrP ORF polymorphisms (homozygous or heterozygous compared to wild type) and the 12 and 23 bp indels (+/+, +/− and −/− compared). All dosed BSE-negative cattle were censored according to their age at cull.

## Results and discussion

Previously published results of the first phase of the study established BSE in all ten cattle dosed with 3×100 g (IP range: 33–45 mpe) and 100 g (IP range: 31–60 mpe), in seven of nine cattle dosed with 10 g (IP range 41–72 mpe, the tenth died of an intercurrent disease at 14 mpe), and in seven of ten cattle dosed with 1 g (IP range: 45–72 mpe) [1]. In the second phase interim published results reported BSE in three of four cattle dosed with 1 g (IP range: 58–73 mpe), in seven of fifteen dosed with 100 mg (IP range: 53–98 mpe) and in single cattle from groups of fifteen dosed with 10 mg (IP: 56 mpe) or 1 mg (IP: 68 mpe).

After publication of the interim findings, two further cases of BSE were diagnosed in cattle in the second phase, one dosed with 100 mg and the other with 10 mg. For completeness of the data from the second phase the times from exposure to onset of the different clinical stages and cull for all BSE-positive cases are given in Table 1 and for all other cattle where BSE was excluded by postmortem tests in Table 2. Neuropathological examination confirmed a vacuolar profile in the brain of the case dosed with 10 mg consistent with that reported previously in the study and with that of naturally affected cattle [1]. The animal dosed with 100 mg and culled with spastic syndrome did not present with vacuolar changes in the brain but in both cases the diagnosis of BSE was confirmed by detection of PrP^d^ immunohistochemically and PrP^res^ on WB.

**Table 1:** Times from exposure to onset of stages and cull in confirmed BSE cases

**Animal identity**	**Possible signs**	**Probable signs**	**Definite signs**	**Cull**
**1 g dose group**				
CM917	**55**	**56**	**58**	59
CN944	**53**	**62**	**64**	65
CM897	**62**	**68**	**73**	73
**100 mg dose group**				
CN938	**49**	**52**	**53**	56
CM900	**49**	**50**	**53**	58
CN951	**58**	**59**	**62**	63
CM909	**45**	**50**	**74**	75
CM936	**62**	––	**75**	77
CM934*	**62**	**74**	**90**	90
CN942	**61**	**97**	**98**	98
CN954*^†^	**50**	**127**	––	127
**10 mg dose group**				
CM898	**49**	**50**	**56**	58
CM923	**62**	**107**	**109**	110
**1 mg dose group**				
CN947	**56**	**62**	**68**	70

All cattle belonged to the second phase of the study.

Times are displayed in months.

* No vacuolar changes in the brain.

^†^ Culled because of spastic syndrome.

**Table 2:** **Times from exposure to onset of stages and cull with experimental outcome in BSE**-**negative cattle**

**Animal identity**	**Possible signs**	**Probable signs**	**Definite signs**	**Cull**	**Experimental outcome**
**1 g dose group**					
CN939	**49**	––	––	53	Spastic syndrome
CM933	**56**	––	––	144*	
**100 mg dose group**					
CN950	––	––	––	97	Urethral obstruction
CM921	––	––	––	100	Hip injury
CN946	**117**	––	––	144	Died, no cause identified
CM906	––	––	––	145*	
CM914	––	––	––	145*	
CN948	––	––	––	145*	
CN949	––	––	––	145*	
**10 mg dose group**					
CN952	––	––	––	84	Indigestion?
CM915	––	––	––	85	Metabolic disease
CM929	**71**	––	––	101	Muscle trauma
CM919	––	––	––	119	Osteoarthrosis
CM931	––	––	––	123	Osteoarthrosis
CN941	**65**	––	––	126	Recumbency, hypophosphataemia
CM904	**53**	––	––	136	Recumbency, thyroid adenoma
CM899	––	––	––	144*	
CM916	**47**	––	––	144*	
CM924	**138**	––	––	144*	Compressive spinal cord lesion
CM913	––	––	––	145*	
CM928	––	––	––	145*	
CN943	––	––	––	145*	
**1 mg dose group**					
CM922	**64**	––	––	81	Renal failure
CM932	––	––	––	103	Died, no cause identified
CM926	––	––	––	104	Urethral obstruction
CN955	––	––	––	109	Died – indigestion?
CM910	**73**	––	––	112	Osteoarthrosis
CM907	––	––	––	123	Spastic syndrome
CM920	**94**	––	––	125	Osteoarthrosis
CM930	––	––	––	136	Recumbency, meningeal tumour
CM905	**62**	––	––	144*	
CN940	––	––	––	144*	
CM896	––	––	––	145*	
CM903	**62**	––	––	145*	
CN937	**106**	––	––	145*	Hippocampal lesion (focal dysplasia)
CN945	**127**	––	––	145*	
**Undosed controls**					
CM908	––	––	––	39	Vertebral fracture
CM918	**62**	––	––	97	Pleural mesothelioma
CM925	––	––	––	59	Femoral fracture
CM856	**61**	––	––	101	Septic arthritis
CM858	**71**	––	––	103	Muscle injury, spastic syndrome
CM901	**56**	––	––	125	Chronic enteritis (Johne’s disease)
CM927	**135**	––	––	144	Found dead (indigestion?)
CM935	**32**	––	––	145*	
CM859	**117**	––	––	145*	
CM902	––	––	––	145*	

All cattle belonged to the second phase of the study.

^*^ Culled because of termination of study.

Times are displayed in months. For controls, months are displayed as months after test group exposure.

The clinical duration in confirmed BSE cases (time from onset of possible signs to cull, excluding those animals that were culled prior to displaying definite signs) ranged from 4 months (CM917 dosed with 1 g) to 48 months (CM923 dosed with 1 mg). Although there was a tendency of cattle exposed to extremely high doses (3× or single dose of 100 g) to have a shorter clinical duration compared to lower doses [median (range; standard deviation) for 3×100 g: 9 (5–18, 4.5), for 100 g: 13 (6–33; 8.7), for 10 g: 18 (11–43; 13.0), for 1 g: 18.5 (4–32; 8.9) and for 100 mg: 15 (5–37; 12.8) months; see [1] and Table 1, the difference was not statistically significant (*P* = 0.08, Kruskal-Wallis test, GraphPad Prism version 5, GraphPad Software, La Jolla, USA). Determination of the exact clinical onset to estimate clinical duration is invariably subjective because it is often based on the display of behavioural changes that may also occur to some extent in “normal” cattle, as observed in undosed controls in this study, even in the absence of another underlying condition. It is for this reason that throughout the study IP has been defined on the basis of onset of definite signs.

No novel polymorphisms were detected in the ORF regions of the 86 tested cattle. Variations in the number of N-terminal octapeptide repeats among PrP gene sequences were reported previously [1]. The other ORF DNA polymorphisms detected were silent in that they do not result in an amino acid change. A summary of the results is given in Table 3. The findings were consistent with those reported previously [5]. Five common promoter genotypes were identified in this study, with the sixth (23 bp −/−, 12 bp +/+) being relatively rare (only found in one animal, see Table 3), which is consistent with previous findings [7]. Proportional hazard regression analysis using the data from 76 orally dosed cattle revealed that the dose was the most important predictor of hazard (*P* < 0.0001), which was expected, whereas none of the genotype parameters were significant predictors (*P* > 0.3). In other words, the main factor that determined how rapidly orally dosed cattle succumbed to BSE was the dose of inoculum, and the genotype did not appear to have a significant influence. Whilst it has been reported that homozygous carriers of the 12 bp insertion allele (12 bp +/+) have a lower risk of developing natural BSE [7], this promoter genotype did not appear to reduce susceptibility to oral exposure to BSE. This may be due to sample size in the current study: to have a reasonable power (80%) to detect the differences (20.5%) as observed by Juling et al. [7], for UK Holstein cattle sample sizes of 60 (12 bp +/+) and 210 (12 bp +/− or −/−) would have been required.

**Table 3:** Bovine PrP genotypes in 86 cattle with numbers of dosed cattle separated by BSE status

**Genotypes**	**Number(Frequency)**	**Number BSE**-**positive dosed**	**Number BSE-negative dosed**	**Total dosed**
**ORF**				
6:6 silent Q78 HET	28 (33%)	9	18	27
6:6 WT	25 (29%)	10	12	22
6:6 silent Q78 HOM	10 (12%)	4	3	7
6:5 silent Q78 HET	7 (8%)	3	4	7
6:6 silent N192 HET	5 (6%)	4	1	5
6:5 WT	4 (5%)	3	0	3
6:6 silent Q78 HET & silent N192 HET	3 (3%)	0	1	1
6:5 silent N192 HET	2 (2%)	0	2	2
5:5* WT	1 (1%)	1	0	1
6:6 silent P113 HET	1 (1%)	1	0	1
**Promoter**				
23 bp +/− 12 bp +/−	31 (38%)	12	17	29
23 bp −/− 12 bp −/−	27 (33%)	10	12	22
23 bp +/+ 12 bp +/+	10 (12%)	5	3	8
23 bp +/− 12 bp +/+	8 (10%)	3	4	7
23 bp −/− 12 bp +/−	5 (6%)	3	2	5
23 bp −/− 12 bp +/+	1 (1%)	1	0	1

Genotypes 5:5, 6:6 and 6:5 refer to the copies of octapeptide repeats, 23 bp and 12 bp are the indels (− = deletion allele, + = insertion allele), Q78, P113 and N192 are silent polymorphisms, either homozygous (HOM) or heterozygous (HET) at position 78, 113 and 192 of the ORF respectively compared to the wild type (WT).

Four of the 86 cattle were excluded from the comparison of the promoter region because of an inconclusive assay result for the 23-bp indel.

* Exposed to 1 g in the first phase of the study and erroneously reported as having a 6:6 genotype previously [1].

**Figure 1:**
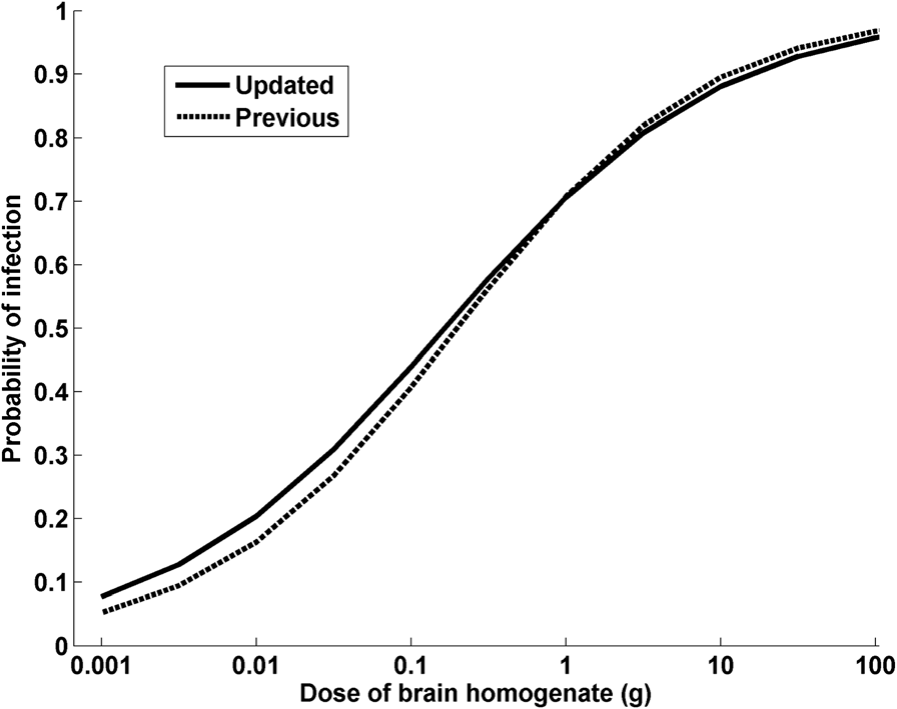
**Estimated probability of infection given the dose of brain homogenate used in the study.** The probability of infection given the dose *S*(*d*) could be expressed as: *S*(*d*) = *exp*(−*3*.*01*+*1*.12*d)/(*1*+*exp*(−*3*.*01*+*1*.12*d)), which results in the updated graph displayed as continuous line. For comparison, the previous graph based on interim data is displayed as dotted line, as published in 2007 [1].

Using the individual IP data for each confirmed BSE case (Table 1 and [1] for phase 1 of the study), the probability of infection given the dose *S* (*d*) could be expressed as: *S*(*d*) = *exp*(*α*+β*d)/(*1*+*exp*(*α*+β*d)) with parameters (and 95% confidence intervals) given by *α* = −3.01 (−3.59, −2.44) and *β* = 1.12 (0.93, 1.33) (see Figure 1), which is a slight modification to the parameters reported previously (*α* = −3.50; *β* = 1.26 [1]), and the additional data resulted in a revised estimate of one cattle oral ID_50_ being equivalent to 10^2.7^ mouse i.c./i.p. ID_50_/g (with 95% confidence interval of 10^2.0^, 10^3.4^) compared to the previous estimate of 10^2.8^ mouse i.c./i.p. ID_50_/g (with 95% confidence interval of 10^2.1^, 10^3.5^). This ID_50_ estimate is equivalent to 0.15 g of the brain homogenate used in the experiment (previous estimate: 0.20 g), with a 95% confidence interval of 0.03–0.79 g. As it has been shown that one mouse i.c./i.p. ID_50_ equals 10^2.7^ cattle i.c. ID_50_[8], it can be extrapolated that one cattle oral ID_50_ equals 10^5.4^ cattle i.c. ID_50_, (previously estimated at 10^5.5^ cattle i.c. ID_50_).

**Figure 2:**
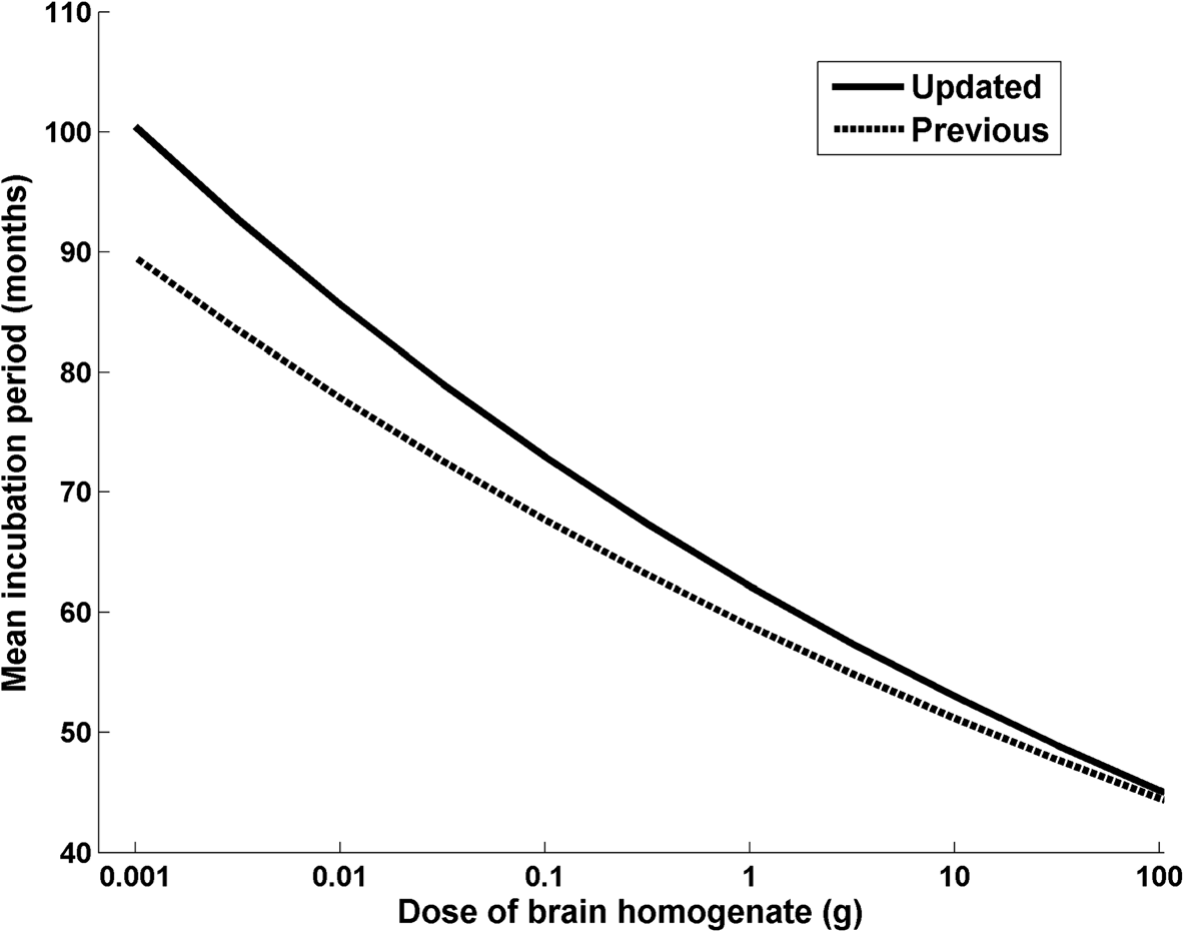
**Estimated mean incubation period according to dose of brain homogenate used in the study.** The updated graph displayed as continuous line is based on the current data. The previous graph based on interim data was published in 2007 [1] and is displayed as dotted line for comparison.

The IP followed a lognormal distribution with parameters of *μ* = a−b×log_10_(dose), with a = 4.66 (4.54, 4.71) and b = 0.14 (0.13, 0.17), and *σ* = 0.23 (previously: *μ* = 4.54−0.14×log_10_(dose), *σ* = 0.21), where dose is the titre of brain homogenate in terms of mouse i.c./i.p. ID_50_/g [see Figure 2, which shows the association between mean IP (given by exp(*μ*+0.5*σ*^*2*^) and the dose].

Preliminary findings from the original study contributed to quantitative risk assessment of the exposure of humans to consumption of infected bovine products [9]. An estimate of human ID_50_ assumed the worst case of a cattle to human species barrier of a factor of one, giving the range of human oral ID_50_s in 1 g of brain from a clinically affected cow as approximately 0.52 to 5. Data from the previously published interim results revised this estimate to 1.0 to 20 and additional results in the present study indicate that this range should now be revised to 1.3 to 33.3, although, as previously, it could be greater with higher titres of BSE affected brain than used in the present study. These estimates have been used to assess the impact of BSE control measures on potential consumption of BSE infectivity (BSE control model [10]). Although the reduced ID_50_ based on the present results would increase estimates of the exposure of humans in terms of bovine oral ID_50_s, the effect would be comparatively small relative to the uncertainty in such risk assessments. Nevertheless, with decline of the BSE epidemic and the potential for relaxation of certain controls, the revised estimate of human oral ID_50_ is available to revisit risk assessments.

The present data do not affect the previous approximation that single doses in the range from 100 mg to 1 g of the brainstem homogenate used correspond to the range of mean IPs of cattle through the BSE epidemic [1]. The observation that a relatively small, single exposure (less than 1 g of high titre brain) can result in infection reinforces the importance of preventing cross-contamination during feed ingredient storage and feed production. This proved to be problematical in feed mills producing ruminant and non-ruminant feedstuffs as is evident from the incomplete effect of the initial statutory control on the feeding of meat and bone meal to ruminants introduced in the UK in 1988. The low dose phenomenon, together with the persistent viability of the BSE agent, has required the removal of specific high risk tissues from cattle at slaughter and the total ban on the use of mammalian meat and bone meal for use in farmed livestock [11].

## Conclusions

The present results concur with the interim findings of this study, that the oral exposure of cattle to BSE brain homogenate produced dose dependent effects on IP and attack rate such that in general the higher the dose the shorter the IPs and the greater the attack rate. In all cases the induced disease closely resembled the pathology of the natural disease. This is in keeping with the analysis of the pathology in orally dosed cattle from another study [12] and reinforces the validity of the oral exposure model for the study of classical BSE in the natural host. The estimate of a cattle oral ID_50_ is revised to 0.15 g brain material used for the studies. Decline of the BSE epidemic indicates that the use of a revised estimate of human oral ID_50_ in risk assessments is, in future, likely to contribute mainly to reassessments in relation to possible relaxation of controls.

## Competing interests

The authors declare that they have no competing interests.

## Authors' contributions

SACH managed the first phase of the study, whilst TK led the second phase of the study and drafted the manuscript, with contributions from MEA, JWW and GAHW. Statistical analyses were performed by MEA with contributions from ARS. Clinical examinations were carried out by TK (second phase) and ARA (both phases); ARA also determined the clinical stages. Genotyping was performed and interpreted by SC. MJS and MMS were responsible for the Western immunoblot and pathological examinations respectively. SACH, JWW, MD and GAHW were involved in the design and initiation of the study. All authors read and approved the final manuscript.
